# Effects of *Bacillus licheniformis* on the growth performance and expression of lipid metabolism-related genes in broiler chickens challenged with *Clostridium perfringens*-induced necrotic enteritis

**DOI:** 10.1186/s12944-016-0219-2

**Published:** 2016-03-08

**Authors:** Mengjia Zhou, Dong Zeng, Xueqin Ni, Teng Tu, Zhongqiong Yin, Kangcheng Pan, Bo Jing

**Affiliations:** Animal Microecology Institute, College of Veterinary Medicine, Sichuan Agricultural University, Chengdu, Sichuan China; Key Laboratory of Animal Disease and Human Health of Sichuan Province, Chengdu, Sichuan China; Animal Genetics and Breeding Institute, College of Animal Science and technology, Sichuan Agricultural University, Chengdu, Sichuan China

**Keywords:** Broiler chicken, Necrotic enteritis, Growth performance, Antioxidant, Lipid metabolism

## Abstract

**Background:**

Necrotic enteritis (NE), caused by *Clostridium perfringens*, has cost the poultry industry $2 billion in losses. This study aimed to investigate the effect of *Bacillus licheniformis* as dietary supplement on the growth, serum antioxidant status, and expression of lipid-metabolism genes of broiler chickens with *C. perfringens-*induced NE.

**Methods:**

A total of 240 one-day-old broilers were randomly grouped into four: a negative control, an NE experimental model (PC), chickens fed a diet supplemented with 30 % of fishmeal from day 14 onwards and challenged with coccidiosis vaccine (FC), and NE group supplied with feed containing 1.0 × 10^6^ CFU/g *B. licheniformis* (BL).

**Results:**

Body weight gain, feed conversion ratio, serum antioxidant status, and lipid-metabolism-gene expression were analyzed. In the PC group, FCR increased significantly whereas serum catalase and glutathione peroxidase activity decreased compared with NC group. Dietary *B. licheniformis* supplementation improved FCR and oxidative stress in experimental avian NE. Using *Bacillus licheniformis* as a direct-fed microbial (DFM) could also significantly upregulate catabolism-related genes, namely, peroxisome proliferator-activated receptor-α and carnitine palmitoyltransferase-1, in livers and changed the expression of lipid-anabolism genes.

**Conclusion:**

These results suggested that dietary *B. licheniformis* supplementation can enhance growth and antioxidant ability, as well as change the expression of genes related to fatty-acid synthesis and oxidation in the livers of NE-infected broilers.

## Background

Necrotic enteritis (NE) is a type of enterotoxemia caused by *Clostridium perfringens* [1], it is a common disease affecting the poultry industry and the high contamination rates of poultry by C. *perfringens* can cause a threat to public health through the food chain [2]. An NE outbreak in broiler chickens often results in high mortality rates and reduced growth performance [3, 4]. Various in-feed antibiotics have been used to prevent and control this disease [4–6]. However, the use of a large amount of antibiotics as growth promoters can cause antibiotic-resistant genes to spread extensively by promoting the selection of antibiotic-resistant bacteria in animals. In view of this concern, many countries have limited the use of non-therapeutic antibiotics in poultry feed; as a result, NE incidences have increased significantly over the past decade [7, 8]. This disease costs the poultry industry $2 billion annually in the purchase of drugs to treat NE and in the lost body weight gain (BWG) [9, 10]. With the banning of in-feed antibiotics use, alternative methods of preventing NE outbreaks must be developed.

A potential approach to NE control is to supplement probiotics in the diets of broiler chickens to manipulate their gut ecosystems. Over the past few decades, *Bacillus* spp*.*, such as *B. licheniformis* and *B. subtilis*, have been used in competitive exclusion experiments. *B. subtilis* competitively exclude *C. perfringens* from broiler chicks; in addition, these bacteria significantly improved body weight and feed efficiency [11–15]. *B. licheniformis*, which are “generally recognized as safe” bacteria, have long been extensively used in the poultry industry. This bacteria can serve as an alternative to antibiotics to enhance growth performance in poultry [16] and is a useful prebiotic for overcoming NE in a commercial-like condition [17, 18].

In broilers, the intermediary metabolism of lipids and energy usually occurs in the liver [19], as does the majority of the de novo fatty acid synthesis process [20, 21]. In the modern poultry industry, chickens are subject to various stress factors that can thus influence lipid metabolism [22–24]. Studies show that the livers of broilers suffering from NE undergo pathological changes [4, 25, 26]; nonetheless, changes in the lipids of broilers infected with *C. perfringens* are rarely investigated. At present, few studies have demonstrated the efficacy of *B. licheniformis* as prophylactic agents against NE in broilers. Therefore, the objectives of our present study are to investigate the effect of *B. licheniformis* on growth performance, on lipid metabolism, and on the hepatic expression of lipogenic genes in broilers infected with NE.

## Results

### Effect of *Bacillus licheniformis* on the growth performance of broilers suffering from necrotic enteritis

The mean values of BWG, feed intake (FI), and feed conversion ratio (FCR) are shown in Table 1. FI did not vary across all of the groups throughout the experimental period; moreover, the *B. licheniformis* supplement group significantly increased BWG and improved FCR in the first two weeks (*P* < 0.05) in comparison with the unsupplemented groups. In the final two-week period of the trial, the BWG of the infected chickens in the NE experimental model group (PC) were significantly lower than those of the negative control group (NC) by 11.5 % (*P* < 0.05). In the same period, FCR impaired by approximately 12.4 points because of the NE infection in the PC group compared with the negative control. The BWG of the infected chickens supplemented with *B. licheniformis* increased significantly over those of the PC group (*P* < 0.05). Moreover, the FCR of the BL group did not differ significantly throughout the final two-week period, from that of the broilers in the NC group.

**Table 1:** The effect of *Bacillus licheniformis* on the growth performance suffering from necrotic enteritis*

Parameter	NC	PC	FC	BL	SEM	*P*-value
Days 1 to 14						
BWG (g)	361.33^b^	358.42^b^	367.13^b^	382.25^a^	4.32	0.026
FI (g)	519.84	521.70	523.92	522.25	2.63	0.194
FCR(g/g)	1.44^a^	1.46^a^	1.43^a^	1.37^b^	0.02	0.049
Days 14 to 28						
BWG (g)	718.87^a^	636.3^b^	689.33^ab^	712.93^a^	10.76	0.023
FI (g)	1159.82	1149.27	1160.83	1161.40	15.41	0.968
FCR(g/g)	1.61^b^	1.81^a^	1.68^ab^	1.63^b^	0.03	0.027
Days 1 to 28						
BWG (g)	1080.20^a^	994.67^b^	1056.67^ab^	1095.27^a^	13.15	0.032
FI(g)	1679.64	1670.90	1684.72	1683.65	15.58	0.991
FCR(g/g)	1.56^b^	1.69^a^	1.60^ab^	1.56^b^	0.02	0.034

*NC* negative control group, *PC* necrotic enteritis experimental model group, *FC* fishmeal and coccidiosis vaccine challenge group, *BL* NE group supplied with feed containing *B. licheniformis*

* Data are means for 5 replicates of 12 broiler chickens presented with the means ± SEM

*^a, b^ Means in the same column with different lower case letter differ significantly (*P* <0.05)

### Effect of *Bacillus licheniformis* on the serum biochemical parameters of broilers infected with necrotic enteritis

**Fig. 1:**
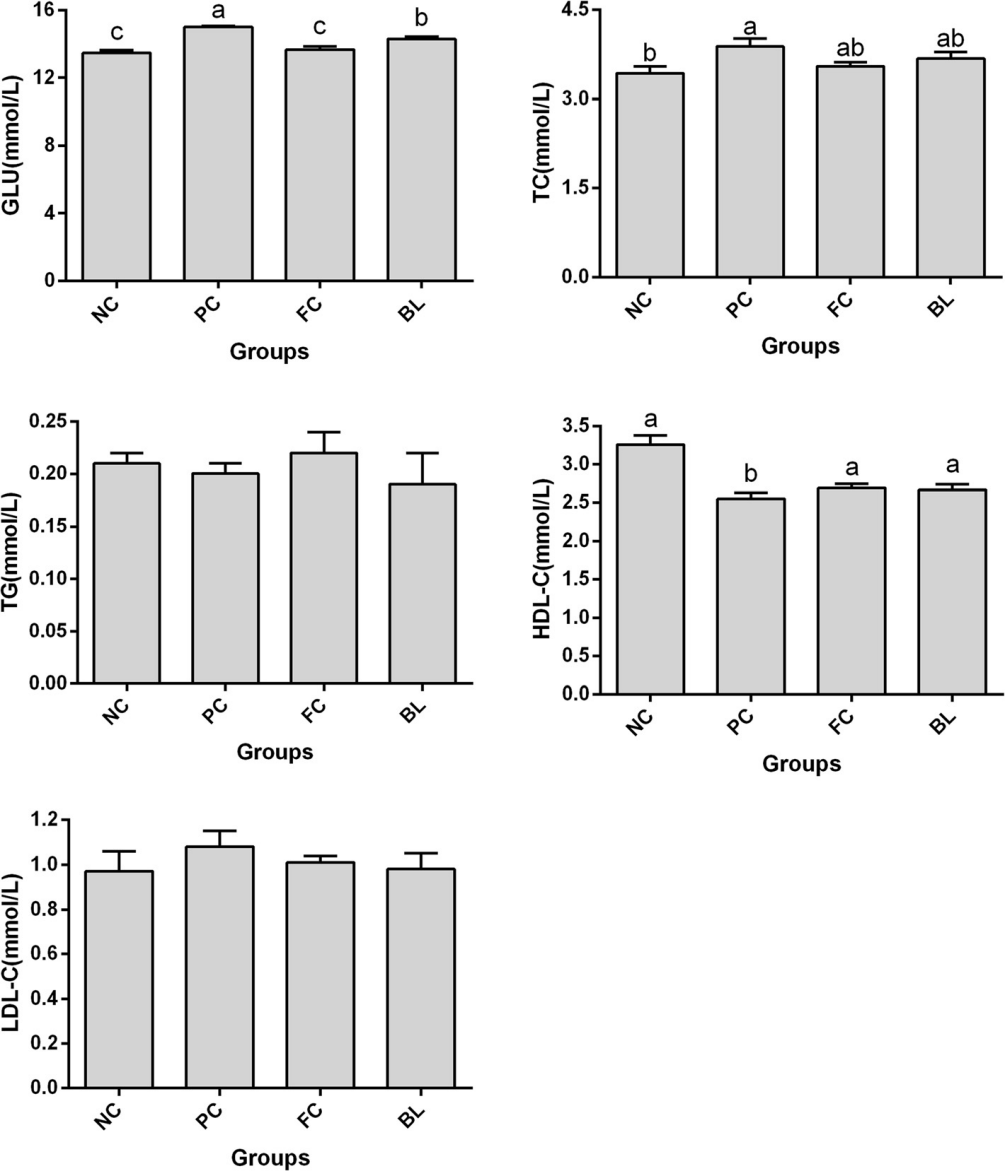
The effect of *Bacillus licheniformis* on serum biochemical parameters of broilers infected with necrotic enteritis. NC: negative control group; PC: necrotic enteritis experimental model group; FC: fishmeal and coccidiosis vaccine challenge group, BL: NE group supplied with feed containing *B. licheniformis.* Data are with the means ± SEM (*n* = 10). ^a-c^ Means with different letter are significantly different (*P* <0.05)

The serum lipid parameters are presented in Fig. 1. The serum levels of triglycerides (TG) and low-density lipoprotein cholesterol (LDL-C) were unaffected by NE infection (*P* > 0.05) in all groups. The NE-infected birds in the PC group exhibited high glucose (GLU) and total cholesterol (TC) levels (*P* < 0.05). The stricken birds in the BL group, which were co-treated with *B. licheniformis* at a dose of 1.0 × 10^6^ CFU/g, displayed a considerably lower serum GLU level (*P* < 0.05) and a significantly higher high-density lipoprotein cholesterol (HDL-C) level than the PC group did (*P* < 0.05). The values of all of the serum lipid statuses of the birds fed with a diet supplement containing 30 % fishmeal and a coccidiosis vaccine (FC group) did not differ significantly from those of NC group.

### Effect of *Bacillus licheniformis* on the serum antioxidant statuses of broilers suffering necrotic enteritis

**Fig. 2:**
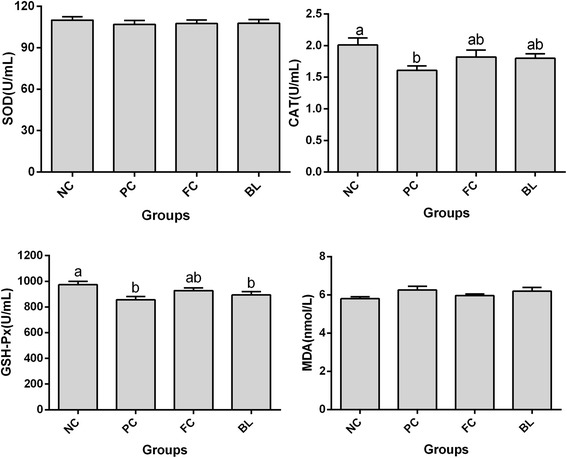
The effect of *Bacillus licheniformis* on the serum antioxidant status of broilers suffering necrotic enteritis. NC: negative control group; PC: necrotic enteritis experimental model group; FC: fishmeal and coccidiosis vaccine challenge group, BL: NE group supplied with feed containing *B. licheniformis.* Data are means for 5 replicates of 12 broiler chickens presented with the means ± SEM (*n* = 10). ^a, b^ Means with different letter are significantly different (*P* <0.05)

The serum antioxidant statuses of the broilers are presented in Fig. 2. Serum superoxide dismutase (SOD) activity did not vary significantly across all of the groups; furthermore, the malondialdehyde (MDA) content in the PC group that was infected with NE and was not supplemented with *B. licheniformis* was higher than that in the NC group, although the difference was insignificant.

**Fig. 3:**
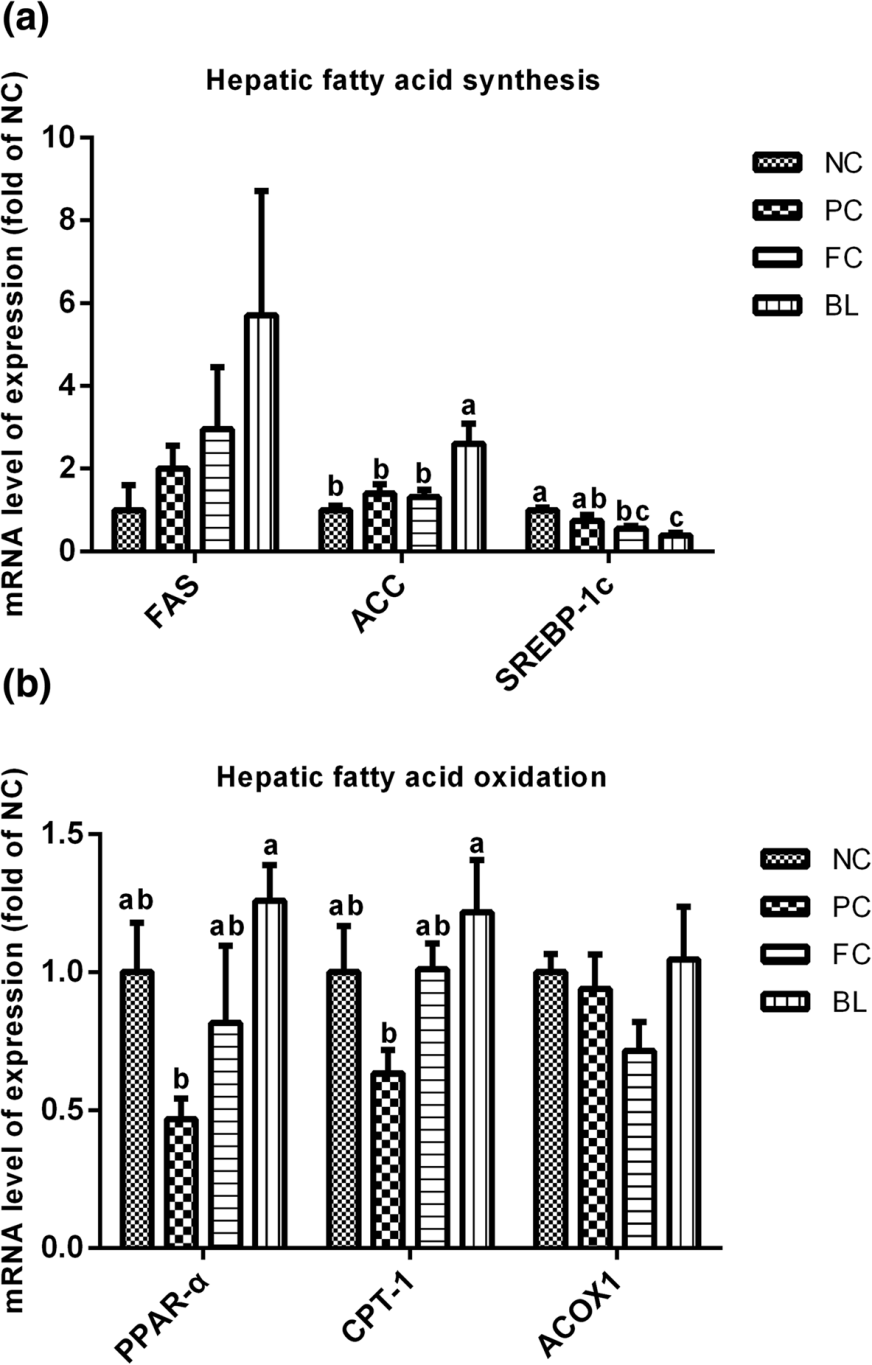
Effect of *Bacillus licheniformis* on the expression of genes related to fatty acid synthesis (**a**) and oxidation (**b**) in the livers of broilers. NC: negative control group; PC: necrotic enteritis experimental model group; FC: fishmeal and coccidiosis vaccine challenge group, BL: NE group supplied with feed containing *B. licheniformis.* Data are the means ± SEM of five chicks in each group. ^a-c^ Means with different letters are significantly different (*P* <0.05)

Serum catalase (CAT) activity was significantly lower in the PC group that suffered from NE and was not supplemented with *B. licheniformis* than in the NC group (*P* < 0.05). The CAT activity of the chickens in the BL group displayed an increasing trend (*P* > 0.05) but did not differ from the NC and PC groups (*P* > 0.05).

NE infection considerably reduced the enzyme activity of glutathione peroxidase (GSH-Px) in both the PC and BL groups in comparison with the NC group (*P* < 0.05). The NC and FC groups did not vary significantly (*P* > 0.05).

### Effect of *Bacillus licheniformis* on the expression of lipid-metabolism genes in the livers of broilers

As shown in Fig. 3, the mRNA level of acetyl-CoA carboxylase (ACC) in the livers of birds in the BL group, who were dosed with 1.0 × 10^6^ CFU/g *B. licheniformis*, was higher than those of the other three groups (*P* < 0.05). In addition, *B. licheniformis* did not significantly affect the expression of the fatty acid synthase (FAS) gene in the liver although the BL group displayed the highest expression (*P* > 0.05) of all of the groups. In addition, sterol regulatory element-binding protein-1c (SREBP-1c) expression was considerably lower in the BL group than in the NC and PC groups (*P* < 0.05).

*B. licheniformis* supplementation can upregulate the expression levels of genes related to fatty acid oxidation. This bacteria significantly increased the mRNA level of carnitine palmitoyltransferase-1 (CPT-1) and enhanced the expression of peroxisome proliferator-activated receptor-α (PPAR-α) in the BL group compared with the PC group (*P* < 0.05). Acyl CoA oxidase 1 (ACOX1) expression did not differ significantly across all groups (*P* > 0.05); nonetheless, that of the BL group was higher than that of the PC group.

## Discussion

In the present study, the growth performance of broilers infected with *C. perfringens* was significantly poorer in the PC group than in the other groups. The reduced BWG and impaired FCR can be indicated by the damaged intestinal mucosa that accompanies *C. perfringens* infection [27, 28], and this damage can affect feed absorption and thus utilization [18].

*Bacillus* sp. microbes are among the most extensively used, direct-fed growth promoters [15]. These bacteria serve as an alternative to antibiotics. In the current experiment, a diet supplemented with *B. licheniformis* can significantly improve BWG and FCR despite *C. perfringens* infection. These effects are mainly attributed to the fact that *B. licheniformis* can enhance nutrient digestion and utilization in broilers by producing several enzymes, such as lipase, protease, and amylase [29, 30]. The diets supplemented with *Bacillus* sp. as a growth promoter can improve BWG and feed efficiency [15, 24, 31, 32]. Nonetheless, the effects of *B. licheniformis* on positive responses to growth performance, such as changes in gut flora, immunity responses, and direct substrate digestion, remain unclear.

The level of fatty acid circulation between the liver and adipose tissue is related to the containment of serum lipids and lipoproteins [33]. Several studies observed that probiotics can significantly reduce these levels in broilers [34, 35]. These results may be attributed to the ability of probiotics to bind cholesterol in guts; probiotics can remove cholesterol through binding onto cellular surfaces [36] and also can convert cholesterol to coprostanol for direct excretion via feces [37, 38]. Increased fecal-lipid and bile-acid output can reduce accumulate serum levels and liver lipids in the body [39].

The present data demonstrate that the birds infected with NE exhibited the highest level of GLU, TC, and LDL-C among those in all of the groups. NE-infected birds that were co-treated with *B. licheniformis* in this study had low concentrations of TC, GLU, and LDL-C but high levels of HDL-C. This result agrees with the finding presented by Yeon *et al*., who suggested that *B. licheniformis* can improve lipid metabolism in mice who were fed a high-fat diet [40].

Researchers hypothesize that oxidative stress influences growth performance and lipid metabolism in animals [15, 24, 41, 42]. In the present study, SOD, CAT, and GSH-Px serum activities were assayed as indices for serum antioxidant capacity, whereas MDA content served as an indicator to determine serum lipid peroxidation levels. MDA is among the most studied products of polyunsaturated fatty acid peroxidation, and its lipid peroxidation is facilitated by reactive oxygen species (ROS). The BL group in the current study displayed a low MDA level, thus indicating that *B. licheniformis* treatment can reduce oxidative stress. The inhibition of SOD, GSH-Px, and CAT activities contributes to the onset of many diseases [43]. In the present study, NE infection lowered CAT and GSH-Px serum activities. This result is similar to that of the research conducted by Lee *et al*., which postulated that SOD, GSH-Px, and CAT activities were inhibited in birds infected with NE [44]. In a previous study, dietary *B. licheniformis* supplementation significantly enhanced the antioxidant capability of triangular breams [45]. In this research, BL group improved the antioxidant capacities in the serum of broilers by increasing GSH-Px and CAT activities and by decreasing MDA compared with the PC group. This result showed that dietary *B. licheniformis* supplementation is an effective strategy to reduce the oxidative stress of experimental avian NE.

The liver is an important organ in which the majority of lipid metabolism occurs. Thus, the gene expression in livers, which can change the capacity of enzymes in relevant metabolic pathways, plays an important role in altering digestive capability [19]. ACC, which is the first key enzyme in fatty acid synthesis and converts acetyl-CoA into malonyl-CoA, plays an important role in the regulation of fatty acid synthesis in animal tissues. This enzyme often combines with FAS as rate-limiting lipogenesis enzyme [46]. In the current study, both ACC and FAS levels increased in three of the groups, especially in the BL group, unlike in the NC group. This phenomenon may be ascribed to the diet change made to build the NE model. Huang *et al*. determined that diets supplemented with 0.5–2 % soy lecithin can significantly increase the expression of FAS and ACC and change the biochemical status of serum lipids [47]. Zhao *et al*. reported that *C. butyricum* supplementation is related to increased FAS and ME activities, enhanced FAS, ME, and ACC mRNA levels in the liver, and increased intramuscular fat content in broilers [48]. In the current research, *B. licheniformis* supplementation can generate the same effect, possibly because *B. licheniformis* can enhance the digestion and utilization of nutrients from a diet containing a high percentage of fishmeal. This diet was used in the NE experimental model and was difficult for broilers to digest. SREBP-1c is a basic/helix-loop-helix/leucine zipper transcription factor that contributes to lipogenic enzyme expression [49]. This transcription factor can bind to the promoters of several lipogenic enzyme genes and induce their expression [50]. Researchers also report that SREBP-1c is a lipogenic nuclear transcriptional regulator that can directly influence the expressions of ACC, FAS, ME, and SCD [51]. In our present study, however, SREBP-1c gene expression decreased significantly in contrast to the expression of lipogenic enzyme genes. This result may be attributed to the fact that fat composition is increasingly exuberant in the subsequent growth stage of broilers [52], especially as the diet changes and probiotics supplementation increases nutrient digestion and utilization in these chickens.

Different diets can alter the expression of PPARs in broiler livers [53]. PPAR-α plays an important role in lipid metabolism; an increase in the expression of this isoform can upregulate the expression of fatty acid catabolism-related genes (CPT-1, ACOX1) and enhance fatty acid β-oxidation [54, 55]. In the present study, *B. licheniformis* can increase both PPAR-α and CPT-1 expression. The expression of these genes was lowest in the NE-infected birds.

In summary, the present study shows that NE infection can change the expression of genes related to fatty acid synthesis and oxidation. *B. licheniformis* supplementation can adjust the levels of these genes by enhancing the expression of fatty acid β-oxidation-related genes to alleviate the negative effects of such infection.

## Methods

### Preparation of culture strains

*B. licheniformis* H2 (CCTCC NO:M2011133) isolated from the ileums of healthy chickens was provided by the Animal Microecological Research Center (College of Veterinary Medicine, Sichuan Agricultural University, Chengdu, China) and cultured at 37 °C at a shaking rate of 180 rpm for 24 h. The culture was centrifuged at 2000 g for 20 min at 4 °C and then resuspended in LB broth containing 1 × 10^9^ colony-forming units (cfu)/mL. The culture was mixed with a basal diet at a level of 1 g/kg (0.1 %, m/m) per day to ensure the viability of bacteria cells throughout the trial period.

A *C. perfringens* type-A strain isolated from a chicken clinically diagnosed with NE was obtained from China Veterinary Culture Collection Center. The strain was cultured in a cooked meat medium at 37 °C under an anaerobic environment; then, the strain was aseptically inoculated into thioglycollate broth overnight at the same temperature and in the same condition.

### Birds and housing

A total of 240 one-day-old broilers with similar body masses (45.35 ± 0.45 g) were purchased from a local commercial hatchery. All of the broilers were randomly divided into four groups, with five replicates per treatment. Each replicate was assigned to a pen (12 chicks per pen). The four groups are as follows: (1) a negative control group fed with corn-soybean meal diet (NC, negative control); (2) an NE experimental model group (PC, positive control); (3) a group that was fed a diet supplemented with 30 % of fishmeal from day 14 onwards and challenged with coccidiosis vaccine (FC, fishmeal and coccidia); and (4) an infected group given a diet supplemented with *B. licheniformis* (BL, *B. licheniformis* at a dose of 1.0 × 10^6^ CFU/g). The composition of an un-medicated corn-soybean meal diet and high fishmeal diet is shown in Table 2. The diets were formulated according to NRC (1994) [56]. Feed and water were provided ad libitum throughout the study, and all chickens were fed in the same house under a relative humidity of approximately 65 %. The temperature was 33 °C in the first week and then decreased gradually to 24 °C by the third week. Lighting was provided 24 h/day. To prevent cross infection, the sides of the pens were composed of metal. Furthermore, the infected groups were kept at least 4 m away from the healthy groups. All experimental procedures were performed in compliance with the laws and guidelines of Sichuan Agricultural University Animal Care and Use Committee.

**Table 2:** Composition of the diet and nutrient levels

Ingredient (g/kg)	Corn-soybean meal diet	High fishmeal diet
Corn	51.64	53.8
Soybean (44.2 % crude protein)	39.6	7.44
Fish meal(62.8 % crude protein)	0.0	30.0
Colza oil	4.3	4.3
Dicalcium phosphate	1.85	1.85
Limestone	1.3	1.3
D,L-Methionine	0.2	0.2
Salt	0.4	0.4
Choline	0.18	0.18
Vitamin Premix^a^	0.03	0.03
Mineral Premix^b^	0.5	0.5
Nutrient Level^c^		
Crude protein	21.17	25.98
Metabolisable energy (MJ/kg)	14.16	14.31
Methionine	0.49	0.95
Lysine	1.03	1.6
Threonine	0.77	0.95
Calcium	1.07	2.11
Total phosphorous	0.71	1.35

^a^Vitamin Premix provided the following per kilogram of complete feed: vitamin A, 50 000 IU; vitamin D_3_, 10 000 IU; vitamin E, 25 IU; vitamin K_3_, 35 mg; vitamin B_3_, 25 mg; vitamin B_2_, 16 mg; vitamin B_6_, 6 mg; vitamin B_1_, 2 mg; vitamin B_12_, 0.03 mg; nicotinic, 25 mg; folic acid, 0.5 mg

^b^Mineral Premix provided the following per kilogram of basal diet: Mn (as manganese sulfate), 60.00 mg; Zinc (as zinc sulfate), 40.00 mg; Cu (as copper sulfate), 8.00 mg;Fe (as ferrous sulfate), 80.00 mg;Se (as sodium selenite), 0.15 mg; I (as potassium iodate), 0.35 mg

^c^Nutrient levels were calculated composition

### Necrotic enteritis infection

The birds were fed with a basal diet from days 1 to 13. From day 14 onward, the diets of all of the birds were changed to the basal diets supplemented with 30 % fishmeal (w/w), except for that of the chickens in the NC group. On day 15, all of the birds, with the exception of those in the NC group, were inoculated with 10-fold coccidiosis vaccine by oral gavage. The birds in the NC group received sterile phosphate buffered saline instead. On days 18, 19, and 20, the birds in the PC and BL groups were individually infected with 1 mL of *C. perfringens* through a plastic tube containing approximately 2.2 × 10^8^ CFU/mL of this bacteria. The feed of the BL group was dosed with 1.0 × 10^6^ CFU/g *B. licheniformis* throughout the experiment, and samples were collected on day 28.

On day 28, 2 birds per pen (10 birds/treatment) were randomly selected and terminated. The blood for the serum samples was sampled and incubated at 37 °C for 2 h and then centrifuged at 2000 g for 15 min. The broilers were then killed by cervical dislocation and necropsied. The liver samples were washed with ice-cold sterilized saline and frozen in liquid nitrogen immediately. The samples were then stored at −70 °C to determine lipid metabolism mRNA.

### Growth performance

The FI and BWG of the chickens in all pens were measured weekly. Moreover, FCR was calculated and adjusted for the dead broilers.

### Determination of serum biochemical values

The serums TC, GLU, TG, HDL-C, and LDL-C were measured on day 28 with a GS200 automatic biochemical analyzer (Shenzhen Genius Electronics Co., Ltd., Shenzhen, China) according to the manufacturer’s instructions.

### Determination of serum antioxidative status

Activity of SOD, GSH-Px, and CAT activity, as well as the MDA content in serum, were assayed with commercially available assay kits (Nanjing Jiancheng Bioengineering Institute) in accordance with the manufacturer’s instructions as indices for serum antioxidant capacities. In brief, the colorimetric method was used to measure CAT activity; SOD activity was calculated based on an auto-oxidant using the hydroxylamine method; GSH-Px activity was assayed according to a 5, 5’-dithiobis (2-nitrobenzoic acid) method; and MDA content was assayed via a thiobarbituric acid method and absorbance measurement at 532 nm.

### Real-time quantitative polymerase chain reaction (RT-qPCR) analysis of gene expression

The total RNA was extracted from liver samples with RNAiso Plus (TaKaRa, Dalian, China) according to the manufacturer’s instructions. RNA quality was tested on 1.5 % agarose gel by electrophoresis, and the quantity of RNA was determined by measuring the absorbance at 260 and 280 nm by using a spectrophotometer (Nanodrop 2000, Thermo Scientific, USA). A first-stand complementary DNA (cDNA) was reversed immediately with 1 μg of total RNA using a Prime Script ^TM^ RT reagent kit (TaKaRa, Dalian, China) according to the manufacturer’s instructions. All cDNA were stored at −70 °C for further use.

RT-qPCR analysis was conducted by using a CFX96 Real-Time PCR detection system (Bio-Rad, Hercules, CA, USA) with a SYBER Premix Ex Taq^TM^ PCR kit (TaKaRa, Dalian, China). The thermocycler protocol was implemented at 95 °C for 5 min, followed by 40 cycles with 15 s denaturation at 95 °C and 30 s annealing/extension at an optimized temperature. Finally, a melt curve analysis was conducted to verify the purity of the PCR products. The gene-related primers are listed in Table 3. In this procedure, glyceraldehyde-3-phosphate dehydrogenase (GADPH) was employed as a house-keeping gene to normalize the expression data. The ΔΔCt method was used to estimate mRNA abundance, and Ct is determined by (Ct, target − Ct, GAPDH) treatment − (Ct, target − Ct, GAPDH) control. All of the samples (*n* = 5) in each group were analyzed in triplicate, and all gene expression results were presented as the fold difference between the NC and the treated groups.

**Table 3:** Primers used for real-time PCR^a^

Gene	Genbank number	Primers position	Primers sequnce (5' to 3')	Annealing temperature(°C)	References
ACC	NM_205505	Forward	AATGGCAGCTTTGGAGGTGT	60.9	[23]
		Reverse	TCTGTTTGGGTGGGAGGTG		
FAS	J03860	Forward	CTATCGACACAGCCTGCTCCT	62.0	[23]
		Reverse	CAGAATGTTGACCCCTCCTACC		
CPT-1	AY675193	Forward	CAATGAGGTACTCCCTGAAA	57.5	[26]
		Reverse	CATTATTGGTCCACGCCCTC		
PPAR-α	AF163809	Forward	TGGACGAATGCCAAGGTC	60.3	[26]
		Reverse	GATTTCCTGCAGTAAAGGGTG		
SREBP-1c	AY029224	Forward	GAGGAAGGCCATCGAGTACA	60.3	[26]
		Reverse	GGAAGACAAAGGCACAGAGG		
ACOX1	NM_001006205	Forward	ATGTCACGTTCACCCCATCC	54.0	[21]
		Reverse	AGGTAGGAGACCATGCCAGT		
GADPH	NM_204305	Forward	GGTGAAAGTCGGAGTCAACGG	58.4	[57]
		Reverse	CGATGAAGGGATCATTGATGGC		

^a^ ACC, acetyl-CoA carboxylase; FAS, fatty acid synthase; CPT-1, carnitine palmitoyl transferase 1; PPAR-α, peroxisome proliferator activated receptor-alpha; SREBP-1c, sterol regulatory element binding protein 1; ACOX1, acyl CoA oxidase 1; GADPH, glyceraldehyde 3-phosphate dehydrogenase

### Statistical analysis

Data were expressed as the mean ± standard error of the mean (SEM). All of these data were analyzed with SPSS Version 20.0 for Windows (SPSS Inc., Chicago, Illinois, USA). A homogeneity test of variance was performed and the results analyzed with one-way analysis of variance. Specific treatments were compared via the least significant difference test at an assigned p-value of < 0.05. Differences between the means were considered significant when P < 0.05.

## Conclusion

The results of our present study showed that dietary *B. licheniformis* supplementation effectively alleviates the negative effects of NE infection. This supplement can also reduce antioxidant stress, enhance growth performance, and adjust the expression levels of certain key genes related to lipid metabolism. Furthermore, the study data may provide a new insight into the prevention and treatment of NE in broilers.
